# Nurse Marion's Romance

**Published:** 1887-04-16

**Authors:** Kate Furley


### Nurse Marion's Romance.

By Kate Furley.

Chapter IV.—The Crisis.

After this day a subtle tenderness diffused itself through the intercourse of nurse and patient. The simplest words meant something more than they did in the talk of daily life. If Marion brought Holtayne his mid-day soup, or altered a pillow, or drew a blind, there was something in his manner of receiving her attention that made it seem almost a caress; and as he grew stronger and was able to talk more of the past—or at least of selected portions of the past—it seemed as if time had been effaced, and there was being re-enacted in the quiet farmhouse the little idyll which had been played ten years ago by shore and sheltered lane at Saxford-by-the-Sea.

Dr. Brandreth, going out and in to see his patient, appeared to observe nothing strange in their demeanour; but was perhaps a little more elaborately professional in his manner to Nurse Marion than was usual with him. She thought that she also was perfectly official in her behaviour; and only Jack, who had an eye for comedy, perceived that the Doctor gave his orders about food and medicine in a tone that faintly indicated pleading, and the nurse answered with an unconscious suggestion of defiance.

"You have given me a pearl of a nurse," said Jack to the Doctor, once, when the latter had come at an unexpected hour, and Marion had gone out for a little. "I never before thought it could be pleasant to be ill, but she has made it so."

"Nurse Marion is careful and attentive, and has had great experience," replied the Doctor, speaking stiffly and without enthusiasm.

"I know she has had experience," Jack answered lightly. "Her father alone—I knew her when she was a girl, and a regular slave to him—gave her trouble and care enough to train a score of nurses. I used to grudge him the attention he got from her; but now I am profiting by it after a fashion."

"You are fortunate to profit by another's pain," remarked the other, still more coldly, but not without a touch of bitter sarcasm; and concluded the interview abruptly.

Jack watched him go with a slight, amused smile. "He makes but a poor actor, that fellow," was his mental comment.

When Marion returned he accosted her with—"Dr. Brandreth's in love with you, Marion, is he not?"

She blushed. "How did you get hold of such an idea?" she asked, with a touch of vexation, not being a woman who liked to parade her conquests.

"I said a word or two about you when he was here a little while ago, and he was so careful to speak of you only as a nurse and not as a woman—and he didn't praise your professional capacity more than he could help—that I could not help guessing that he was pretty considerably gone on you. When a middle-aged man falls in love, you always guess the depth of his affection by what he leaves unsaid, rather than by what he says. And besides, he went off in such a hurry that he forgot his stethoscope. There it is lying on the table."

Marion removed the stethoscope to the chimney-piece, and said, standing with her back to her patient, "You have so little to interest you here that you imagine a great deal from a very small foundation. I must get some books for you. Do you like novels best?"

"Don't trouble; this little romance affords me quite sufficient excitement. Tell me, has he proposed to you yet?"

"Mr. Holtayne, you have no right—"

"Nurse Marion—since that's your proper title—have I not? And Marion—who was my Marion long ago—can you not understand why I want to know if you are likely to become Dr. Brandreth's wife?"

Jack spoke in the tender accents, just faintly touched with mirth, which had never failed to move Marion. Involuntarily her voice softened as she answered:

"You know that I am not likely to do so."

"Now, Marion, just come here and sit by my side and tell me the whole story. I want something to amuse me."

She took her usual place by his bed, but said, as she did so, "Do not ask me any questions. I can tell you nothing."

"But why?"

"It is Dr. Brandreth's secret, not mine."

"And she does not see," Jack confided to the walls, "that she has betrayed the whole of it by that remark. Now that I know my surmise was correct," he went on, "tell me the whole truth. Has Dr. Brandreth asked you to marry him?"

"Yes," she answered, feeling herself grow weaker under the beseeching look Jack could put into (at will) his dark eyes.

"Have you accepted him?"

"No; of course not."

"That is good news. But has he taken your rejection as final?"

"He has not."

"Ah! then there's no knowing what the end may be. I may have to offer you my congratulations yet."

"You know you will not," cried Marion, in a voice that showed how the suggestion wounded her.

"Are you sure?" asked Holtayne, with gleaming eyes.

"Do you doubt it?" she retorted.

He half raised himself up in bed—he was getting stronger now—and said, "Come nearer, Marion."

She bent forward till his outstretched arms rested on her shoulders. She felt them tremble, and put out her own to support him.

"Ah! my dear, it is a long time since you put your arms around me thus," he said; and then went on: "Let me look at your face. It has not changed much since we parted. Your cheeks are thinner than they were, and there is just a faint line below the eyes to tell of toil and trouble; but the eyes themselves are as bright and clear, and the smile as sweet as ever. Your hair,—you had lovely hair, I remember. I never saw real chestnut hair but yours; but I can't see it now: it is hidden out of sight."

With one hand he unfastened the cap she wore, and threw it impatiently on the floor, uncovering a mass of waving nut-brown hair; but—"Why, there are grey threads in it!" he exclaimed.

Marion smiled sadly. "I am not young now, Jack. I am thirty, and feel older—old enough for all my hair to be grey."

"True, and I suppose I am not young, either. We are both too old for folly, and yet—for the sake of old days, dear, when we were young and happy—kiss me once."

The spell of the past, the memory of the unforgotten days of happiness which had held their charm during all the dreary years that lay between then and now, was upon Marion.

She bent forward, and their lips met.

And this was the very moment selected by Dr. Brandreth to come back for his stethoscope.

Dr. Brandreth started when he saw the attitude of the two occupants of the room; while Holtayne fell back on his pillow, filled with a sense of triumph, though striving to keep within decorous bounds a longing to laugh aloud at the look of pain which crossed the Doctor's face, and Marion stood up and faced the new-comer, pale with emotion and trembling with a mixture of vexation and shame, blended with a sorrow at having caused any grief to the man whose love she had refused, which even at that moment bewildered her by its strangeness. Why should she care? she asked herself. She had no affection for Dr. Brandreth, beyond a kindly liking for him as friend and fellow-worker; she had already refused to accept him as her husband, and he had every reason to be sure that the postponement of her final reply meant no concession to his wishes. Yet when she saw that he was wounded by the evident revival of her old love for Jack Holtayne she felt that she owed him some reparation, and that she would gladly do almost anything to atone to him for the pain he seemed to feel.

The Doctor made no direct comment on the scene he had witnessed.

"I forgot my stethoscope," he explained to Holtayne, in the quietest of tones; but there was some evidence of restrained anger in his voice when he added, turning to Marion, "You have forgetten, I think, nurse, that you are expected to wear your entire uniform when you are on duty, though you are not actually within the walls of the hospital."

Marion picked up her cap from the floor, with a scarlet flush staining the paleness of her cheeks; but she remained silent, though many words rose to her lips, for they were incoherent, and she felt that if she tried to utter them tears must come as well. Before, however, she could regain composure the Doctor had left the room.

"Well, this is rather a joke," observed Jack, laughing openly, now that his rival was gone.

"A joke!" echoed Marion in a tone of vexation; "I wish anyone on earth had come in rather than he."

"I see, mademoiselle; you want to continue your little flirtation with him in spite of the complications introduced by my presence. Well, I shall be generous and not mind—if he is still willing to go on after the little *contretemps* of to-day."

"Jack!" cried the nurse, indignantly, "you know that is not what I mean. But what he saw pained him; you must have observed that yourself."

"I did; in fact, it was obvious to the meanest capacity, and, on the whole, I don't wonder at it."

"In my position, too," she went on, in her self-reproach,—"a nurse who ought to be absorbed in her work—"

"My dear girl, there never yet was a woman who was so absorbed in any work that she had not time to listen to Love's voice when he came to talk to her. And you know it isn't quite as if you were showing tenderness to a stranger. A kiss given to an old lover 'just for the old love's sake' is not such a serious crime that it cannot be forgiven, even by an amorous, not to say jealous, doctor. Explain to him that you were moved only by pity for my forlorn condition, and he'll see the matter in a proper light at once."

"How can you take it so lightly! It is not a thing to make a jest of, wounding such a man as that."

"He holds such a high place in your esteem, then?" said Jack, with a touch of jealousy in his voice.

"Yes," answered Marion, with the firmness of indignation. "I do not care for him as he does for me, but I respect and admire him more than any man I have ever known. It is an honour to me that he should have wished to make me his wife, and not even you shall jeer at him in my presence."

She turned away and occupied herself in putting on her cap, while Jack drummed a faint tattoo on the coverlet, as he studied her profile with a glance in which admiration and irritation were mingled in equal proportions.

The silence which thus remained between them had not been broken, when a knock came to the door. Marion opened it and saw the farmer's wife, who came to tell her that Dr. Brandreth wished to speak to her, and was now waiting for her in the best parlour.

"What does this mean?" asked Holtayne, when the woman had gone, after casting a glance of mystified inquiry at the two occupants of the room, the atmosphere of which seemed even to her not very acute perceptions to be charged with emotion.

"It means," answered the nurse, "that Dr. Brandreth is seriously angry at my conduct."

"I see; jealousy taking the form of official reprobation! And what will his anger end in?"

"I cannot say. Perhaps only in a reproof; perhaps in dismissal."

"Dismissal! By Jove, that's serious," said Jack thoughtfully; "I didn't think there was any risk of that."

Marion waited for a moment, thinking that he would add some more kindly word—some suggestion that the consequences of dismissal from St. Lazarus' might not be so serious to her now, as they would have been under other circumstances. But no such thought seemed to enter his mind, or at least none such appeared to him to need expression. As he saw her hesitating, he remarked, in a tone of vexation:

"I suppose you had better go and get it over at once," and gave his pillow an impatient shake, preparatory to settling himself more comfortably upon it.

*(To be continued.)*

